# Integrating kidney function assessment into the clinical interpretation of plasma Alzheimer's disease biomarkers

**DOI:** 10.1177/13872877251375101

**Published:** 2025-09-09

**Authors:** Burak Arslan, Henrik Zetterberg, Nicholas J Ashton

**Affiliations:** 1 Department of Psychiatry and Neurochemistry, Institute of Neuroscience & Physiology, the Sahlgrenska Academy at the University of Gothenburg, Mölndal, Sweden; 2 Wisconsin Alzheimer's Institute, School of Medicine and Public Health, University of Wisconsin, Madison, WI, USA; 3 Clinical Neurochemistry Laboratory, Sahlgrenska University Hospital, Gothenburg, Sweden; 4 Department of Neurodegenerative Disease, Institute of Neurology, University College London, London, UK; 5 UK Dementia Research Institute, University College London, London, UK; 6 Hong Kong Center for Neurodegenerative Diseases, Hong Kong, China; 7 Centre for Brain Research, Indian Institute of Science, Bangalore, India; 8 Banner Alzheimer's Institute and University of Arizona, Phoenix, AZ, USA; 9 Banner Sun Health Research Institute, Sun City, AZ, USA

**Keywords:** Alzheimer's disease, chronic kidney disease, kidney impairment, p-tau217

## Abstract

As plasma biomarkers like p-tau217 move towards clinical use in Alzheimer's disease (AD), it is important to understand how kidney function may influence their accuracy. Even mild chronic kidney disease (CKD) can alter biomarker levels, potentially impacting test performance. While accounting for renal function may improve specificity, it could reduce sensitivity without greatly changing overall diagnostic accuracy. Most studies focus on mild CKD, leaving gaps in understanding severe CKD—especially in real-world settings like primary care. Including renal indices such as eGFR in diagnostic models could help improve interpretation and minimize misclassification in older adults, where CKD is common.

Impaired kidney function, whether measured using the gold standard iohexol clearance^1^ or estimated glomerular filtration rate (eGFR) based on serum or plasma creatinine and/or cystatin C measurements,^2^ has been shown to affect absolute plasma concentrations of Alzheimer's disease (AD) biomarkers, including phosphorylated tau (p-tau) species, neurofilament light (NfL), and glial fibrillary acidic protein (GFAP).^1–7^ Since impaired kidney function can disrupt the equilibrium of substances in the bloodstream, it may also affect the levels of other routinely measured proteins, such as β2-microglobulin and free immunoglobulin light chains, whose concentrations can change when renal clearance is reduced.^8^ While early studies primarily examined the relationship between serum creatinine and AD biomarkers,^3^ more recent investigations have increasingly relied on eGFR,^2,4,7^ with only a limited number employing iohexol clearance to validate that these biomarkers are elevated in the presence of renal dysfunction.^1^ As variations in kidney function can alter biomarker concentrations, they may also influence diagnostic performance—often leading to reduced sensitivity and increased specificity when adjustments for renal function are applied.^9^ These effects are particularly important to consider in clinical contexts, where accurate interpretation of biomarker levels at the individual level must account for both central nervous system pathology and peripheral factors, such as renal clearance, that can influence blood biomarker concentrations.

The impact of kidney impairment on the performance of blood-based AD biomarkers is particularly relevant in community settings, where the prevalence of chronic kidney disease (CKD) increases with age.^10^ This is a critical consideration when diagnosing sporadic AD and underscores the real-world implications of renal dysfunction on the interpretation of high-performing biomarkers such as plasma p-tau217. Most studies on AD and blood-based biomarkers have been conducted in well-characterized research cohorts, which often exclude individuals with overt renal impairment, thereby limiting the generalizability of findings to broader clinical populations. In contrast, clinical settings that include patients undergoing hemodialysis^1,11^ or those with end-stage renal disease^12^ likely exhibit more pronounced alterations in biomarker levels due to impaired renal clearance. Several studies have investigated the relationship between kidney function and AD blood biomarkers, primarily in individuals with mild-to-moderate CKD.^2–4^ While these studies have shown that absolute concentrations of biomarkers such as p-tau217 vary across CKD stages, mild renal impairment does not appear to significantly affect the amyloid prediction models. However, the underrepresentation of individuals with more advanced CKD may result in an underestimation of the true impact of renal dysfunction. A more recent study,^7^ which included a higher proportion of participants with CKD stages 3–4 (nearly 50% of the cohort), demonstrated that significant renal impairment (CKD stage 3b or higher) may reduce the clinical utility of plasma p-tau217. Interestingly, this effect could be mitigated by using ratios such as the p-tau217/non-phosphorylated tau217 percentage,^4,7^ which may help normalize biomarker levels in the context of reduced renal filtration. The same study^7^ also supported the use of log–log linear models to better capture the nonlinear relationships between eGFR and small proteins such as creatinine and cystatin C. Their findings showed that the increase in p-tau217 was more pronounced at lower eGFR values—for instance, the concentration rise from 45 to 30 mL/min/1.73 m² was greater than that from 60 to 45, highlighting the utility of nonlinear modeling in this context. Additionally, an increased rate of false-positive p-tau217 results was observed as eGFR declined,^7^ reinforcing the potential for diagnostic misclassification in individuals with impaired kidney function. These insights underscore the importance of incorporating eGFR into biomarker-supported diagnostic algorithms, as severe kidney dysfunction (CKD stage 3b or higher) may alter patients’ risk classification based on plasma p-tau217 and necessitate confirmatory testing, such as cerebrospinal fluid (CSF) analysis or positron emission tomography (PET) imaging, to ensure accurate assessment of amyloid status, highlighting the need for further research in this area.

Adding to the growing body of evidence, Sato et al.^9^ recently investigated the impact of adjusting for renal function on the performance of blood-based AD biomarkers, specifically phosphorylated tau species (p-tau181 and p-tau217), measured across multiple immunoassay platforms. Their analysis used data from two cohorts: the PAD-TRACK study, an ongoing trial-ready Japanese cohort, and the ADNI study. Notably, approximately 30% of participants in their combined sample had an eGFR <60 mL/min/1.73 m². The authors incorporated renal function corrections into amyloid PET prediction models and, consistent with previous findings, observed that absolute concentrations of p-tau181 and p-tau217 differed by CKD stage. After correcting for renal function, sensitivity decreased while specificity increased. However, the overall improvement in amyloid prediction accuracy was negligible, which may be partly due to the low proportion of participants with severe CKD in their dataset.

This study adds an important dimension to prior work by not only reaffirming the effect of renal function on biomarker concentrations but also evaluating its influence on sensitivity and specificity. Although the overall predictive accuracy did not change significantly, several practical implications emerge. First, the prevalence of CKD in real-world clinical populations can be much more variable, and often higher, than in clinical trials or research studies. Second, in younger or asymptomatic middle-aged individuals, the influence of renal dysfunction on blood biomarker levels may be minimal; however, the prevalence of CKD in the target population still warrants consideration. Finally, if high-performing blood-based biomarkers such as p-tau217 are to be implemented in primary care settings, where renal impairment may be overlooked, and CKD itself is a known risk factor for cognitive decline,^10^ it becomes crucial to include relevant covariates such as age, creatinine, and potentially other medical conditions like cardiovascular disease in biomarker-supported diagnostic algorithms. This approach could help ensure the accuracy and generalizability of biomarker-based screening tools across diverse patient populations.

It is also important to emphasize that, in routine clinical practice, GFR is estimated using serum creatinine (eGFR_cr_), cystatin C (eGFR_cys_), or a combination of both (eGFR_cr−cys_). However, these different estimation methods often yield divergent results.^13^ Increasing evidence suggests that cystatin C may offer a more accurate assessment of kidney function and a closer approximation of measured GFR. For example, unlike creatinine, cystatin C is minimally influenced by muscle mass. Moreover, it has been suggested that combining both creatinine and cystatin C to estimate GFR (eGFR_cr−cys_) provides the most accurate estimation method across both younger and older adults at the population level.^14^ With this in mind, another intriguing condition, shrunken pore syndrome (SPS),^15^ is characterized by a reduced eGFR_cys_/eGFR_cr_ ratio, typically below 0.60 or 0.70, in the absence of non-renal influences on cystatin C or creatinine. SPS is a kidney disorder marked by a selective reduction in the glomerular filtration of mid-sized molecules (5–30 kDa), such as cystatin C, while smaller molecules (<0.9 kDa), like creatinine, remain relatively unaffected. This impaired filtration leads to decreased clearance of larger molecules, resulting in discrepancies between cystatin C– and creatinine-based eGFR estimates, and may potentially influence the plasma dynamics and interpretation of AD biomarkers. Overall, these findings need to be replicated and validated in future studies, particularly in older populations, to ensure a more accurate estimation of measured GFR.

Taken together, with recent advancements in blood-based AD biomarkers, particularly p-tau217, and the recent FDA approval of the first blood test for AD (based on the p-tau217/Aβ_42_ ratio^16^), the next logical step is clinical implementation, especially in primary care settings. This process would be facilitated by high-throughput immunoassay platforms that are compatible with routine clinical chemistry tests, allowing for seamless integration into standard laboratory workflows. Measuring p-tau217 alongside commonly ordered tests, such as creatinine and cystatin C, would enable adjustment for renal function, which is particularly relevant in aging populations. Alternatively, a more practical approach would be to use eGFR to identify cases where a blood test for AD is likely to be unreliable, and, in those instances, order more advanced diagnostic tests, such as PET imaging or CSF analysis. Moreover, incorporating additional variables, including age, liver function tests, and other potential confounders, into biomarker-supported diagnostic algorithms could enhance the accuracy and clinical applicability of blood-based biomarker testing in real-world settings.
